# Reduced cardiovascular reserve capacity in long-term allogeneic stem cell transplant survivors

**DOI:** 10.1038/s41598-023-28320-w

**Published:** 2023-02-06

**Authors:** Hayley T. Dillon, Stephen Foulkes, Yuki A. Horne-Okano, David Kliman, David W. Dunstan, Robin M. Daly, Steve F. Fraser, Sharon Avery, Bronwyn A. Kingwell, Andre La Gerche, Erin J. Howden

**Affiliations:** 1 Baker Heart and Diabetes Institute, Melbourne, Australia; 2 Institute for Physical Activity and Nutrition, School of Exercise and Nutrition Sciences, Deakin University, Geelong, Australia; 3 University of Melbourne, Melbourne, Australia; 4 Malignant Haematology and Stem Cell Transplantation Service, Alfred Hospital, Melbourne, Australia; 5 CSL Ltd, Melbourne, Australia; 6 Cardiology Department, St Vincent’s Hospital Melbourne, Fitzroy, Australia

**Keywords:** Haematopoietic stem cells, Cancer, Cardiovascular diseases, Haematological diseases, Cancer, Cardiology, Oncology, Risk factors

## Abstract

Premature cardiovascular mortality is increased in long-term allogeneic stem cell transplant (allo-SCT) survivors, but little information exists regarding *subclinical* cardiovascular dysfunction in this population. We compared peak oxygen uptake (${{\dot{\mathrm V}}}$O_2peak_), a prognostic cardiovascular marker, and its determinants between long-term allo-SCT survivors and non-cancer controls. Fourteen allo-SCT survivors (mean ± SD, 44 ± 15 years, 50% male, median time since allo-SCT: 6.5 years [range 2–20]) and 14 age- and sex-matched controls (46 ± 13 years, 50% male) underwent cardiopulmonary exercise testing to quantify ${{\dot{\mathrm V}}}$O_2peak_. Resting echocardiography (left-ventricular ejection fraction and strain), exercise cardiac MRI (peak cardiac and stroke volume index [CI_peak_, SVI_peak_]), biochemistry (hemoglobin, troponin-I, B-natriuretic peptide), dual-energy x-ray absorptiometry (lean [LM] and fat [FM] mass, percent body fat [%BF]) and *Fick*-*principal* calculation (arteriovenous oxygen difference) were also performed. Survivors exhibited impaired ${{\dot{\mathrm V}}}$O_2peak_ as compared with controls (25.9 ± 5.1 vs. 33.7 ± 6.5 ml kg^−1^ min^−1^, *p* = 0.002), which coincided with reduced CI_peak_ (6.6 ± 0.8 vs. 8.6 ± 1.9 L min^−1^ m^−2^; *p* = 0.001) secondary to reduced SVI_peak_ (48 ± 4 vs. 61 ± 8 ml m^−2^; *p* < 0.001) rather than chronotropic impairment, and higher %BF (difference, 7.9%, *p* = 0.007) due to greater FM (5.8 kg; *p* = 0.069) and lower LM (4.3 kg, *p* = 0.25). All other measures were similar between groups. Despite comparable resting cardiac function and biomarker profiles, survivors exhibited reduced ${{\dot{\mathrm V}}}$O_2peak_ and exercise cardiac function and increased %BF relative to controls. These results highlight potential therapeutic avenues and the utility of exercise-based cardiovascular assessment in unmasking cardiovascular dysfunction in allo-SCT survivors.

## Introduction

Allogeneic stem cell transplantation (allo-SCT) is a treatment for several high-risk and recurrent hematological malignancies. Advances in transplant strategies and supportive care have improved cancer-specific survival rates^1,2^. Despite this, cure of the underlying malignancy does not guarantee complete restoration of health. Indeed, consequent to the compounding effects of therapeutic exposures, treatment complications, and unfavourable lifestyle changes in the pre-, peri- and post-transplant period, allo-SCT survivors are 5.7-times more likely than their age- and sex-matched non-cancer counterparts to develop a severe or life-threatening condition^3^.

Cardiovascular disease (CVD) is a leading cause of morbidity and mortality in long-term allo-SCT survivors and, as such, has become a key therapeutic focus to optimise the balance between acute cancer management and long-term health^4,5^. Compared to the general population, allo-SCT survivors have an increased risk of CVD^6–9^, serious cardiovascular events^7,8,10^, and ultimately, premature cardiovascular death^8,11^. Of concern, the long latent period preceding clinically overt CVD suggests the cardiovascular burden among long-term allo-SCT survivors may be underestimated^12^. On the other hand, this highlights an important window of opportunity to detect and address dysfunction at a subclinical stage of disease when targeted interventions may be efficacious. We have recently documented subclinical impairments in cardiac function and skeletal muscle oxygenation as early as 3 months following allo-SCT^13^. However, there is little information regarding subclinical cardiovascular dysfunction among long-term survivors, and so it is unclear whether these immediate impairments are transient or sustained. To better inform therapeutic approaches throughout the survivorship continuum, it is imperative to thoroughly characterise the cardiovascular health of long-term allo-SCT survivors using sensitive assessment tools capable of unmasking subclinical cardiovascular dysfunction.

Cardiovascular reserve capacity (assessed using cardiopulmonary exercise testing [CPET] and quantified as peak oxygen uptake [${{\dot{\mathrm V}}}$O_2peak_]) provides an integrated measure of cardiac, pulmonary, vascular, hematologic, and skeletal muscle function^14^. Each of these components of the oxygen transport and utilisation pathway are at risk of dysfunction among recipients of allo-SCT^15^. In addition, ${{\dot{\mathrm V}}}$O_2peak_ is an established prognostic marker for CVD and may be a more sensitive marker of cardiovascular impairment than traditional single organ resting measures in patients undergoing treatment for cancer^16–20^. Therefore, assessment of ${{\dot{\mathrm V}}}$O_2peak_ and the key organ systems underpinning ${{\dot{\mathrm V}}}$O_2peak_ may provide clinically valuable insight into the etiology of cardiovascular dysfunction among long-term allo-SCT survivors, and aid in the earlier identification and protection of patients at high risk of developing CVD.

Despite its prognostic importance and utility as an early diagnostic measure, ${{\dot{\mathrm V}}}$O_2peak_ and its central and peripheral determinants have not been completely characterised in long-term allo-SCT survivors. In the two studies that have sought to characterise ${{\dot{\mathrm V}}}$˙O_2peak_ in allo-SCT survivors, significant impairment has been observed^21,22^. However, in this population, investigations into the key organ systems underpinning impaired ${{\dot{\mathrm V}}}$O_2peak_ (1) produced conflicting results, and (2) were limited to standard-of-care resting measures of cardiac (i.e., echocardiography) and pulmonary (e.g., spirometry) function which are likely to miss subtle impairments that, in the case of the heart, could be unmasked during exercise (i.e., exercise cardiovascular magnetic resonance imaging [ExCMR])^13,20,23,24^. Further, the potential contribution of vascular, skeletal muscle and body composition metrics to poor ${{\dot{\mathrm V}}}$O_2peak_ is yet to be elucidated among long-term allo-SCT survivors. Therefore, to address these gaps in knowledge, this matched case–control cross-sectional study aimed to compare ${{\dot{\mathrm V}}}$O_2peak,_ and its cardiac, hematological, vascular, and skeletal muscle determinants (using sensitive exercise-based measures) among long-term allo-SCT survivors and an age- and sex-matched non-cancer control group. A secondary aim was to assess whether central factors (cardiac function) or peripheral factors (oxygen carrying capacity, peripheral oxygen extraction, skeletal muscle mass) were associated with ${{\dot{\mathrm V}}}$O_2peak_ in long-term allo-SCT survivors.

## Methodology

### Population and design

This is a matched case–control cross-sectional study that included 14 long-term adult survivors of allo-SCT and 14 age- and sex-matched non-cancer controls. The long-term allo-SCT survivor cohort were recruited from the Alfred Hospital Hematology Department’s Late Effects Clinic and the Royal Melbourne Hospital, and comprised individuals who received allo-SCT for hematological malignancy and had survived ≥ 2 years. Age- (± 2 years) and sex-matched controls were recruited from the community through advertisements seeking ostensibly healthy untrained adults. Participants were excluded if they were aged < 18 years, unable to speak or understand English, had known contraindications to CMR, or were unable to perform cycling exercise. With the interest of discerning how the cardiovascular impairment experienced by allo-SCT survivors compares with ‘normal cardiovascular aging’, additional exclusion criteria were employed for the control group to minimise confounders. These included body mass index ≥ 35 kg m^−2^, significant known medical conditions (e.g., known structural heart disease including symptomatic ischemic heart disease, significant valvular disease or inherited cardiomyopathies), and exceeding current physical activity guidelines of ≥ 150-min of moderate intensity or ≥ 75-min of vigorous intensity aerobic physical activity (assessed via verbal self-report)^25^.

### Experimental measurements

All participants underwent a physiological testing at the Baker Heart and Diabetes Institute clinical research facility in Melbourne, Victoria, Australia. Testing was conducted over two visits, within one week. Participants were asked to refrain from moderate-to-vigorous intensity physical activity (exercise) in the 24-h preceding testing and alcohol and caffeine on the day of testing.

*Medical History.* For the survivor cohort, clinical information including underlying cancer diagnosis, previous cancer treatment, allo-SCT (donor relation, graft source, conditioning intensity/regimen, graft-versus-host-disease [GvHD] status, time since transplantation), current medications, traditional cardiovascular risk profile (e.g., diabetes, overweight/obesity, hypertension, hyperlipidemia) and history of overt CVD and related events (e.g., heart failure, coronary heart disease, peripheral artery disease, myocardial infarction, stroke) were obtained from hospital records at the time of study visit (median of 6.5 years post allo-SCT). Documented CVD and related events were stratified according to time of development/occurrence (e.g., pre-SCT or post-SCT). A general lifestyle questionnaire was administered to the age- and sex-matched non-cancer control group to obtain information relating to current health status (including cardiovascular risk profile), relevant medical history, and use of cardiovascular medications.

*Cardiopulmonary Fitness.* A ramp protocol CPET was conducted on an electronically-braked cycle ergometer (Lode Excalibur Sport, Groningen, the Netherlands) with breath-by-breath expired gas analysis (Jaeger™ Vyntus™ CPX, Vyaire Medical, Mettawa, IL, USA) and continuous heart rate monitoring (Vyntus™ ECG 12-lead PC-ECG, Vyaire Medical, Mettawa, IL, USA) and pulse oximetry (Nonin Medical, Inc, Plymouth, MN) to quantify ${{\dot{\mathrm V}}}$˙O_2peak,_ as well as peak heart rate (HR), oxygen pulse and arterial oxygen saturation. Brachial blood pressure was measured at 2-min intervals using an automated ECG-gated cuff (Tango M2 Stress Test Monitor and Orbit-K Blood Pressure Cuff, Suntech Medical Inc. Morrisville, NC, USA). Cycling commenced at 10–30 Watts for 1-min and increased by 10–30 Watts min^−1^ until volitional fatigue. Tests were deemed a peak effort if participants reached volitional fatigue and met at least one of the following: respiratory exchange ratio ≥ 1.10 or peak heart rate (HR) > 85% age predicted. ${{\dot{\mathrm V}}}$O_2peak_ was defined as the average of the six consecutive highest 5-s VO_2_ values. Age-, sex-, bodyweight-, and height- predicted ${{\dot{\mathrm V}}}$O_2peak_ was calculated using the FRIEND equation^26^.

*Resting Cardiac Function.* Resting cardiac function (left-ventricular ejection fraction [LVEF], and global longitudinal strain [GLS]) was evaluated via echocardiogram (Vivid E95, GE Vingmed, Horten, Norway) as previously described^13^, with images saved in a digital format for offline analysis (Echopac v13.0.00, GE, Norway).

*Cardiac Reserve and Peak Cardiac Function.* The biventricular response to exercise was evaluated using a validated real-time ExCMR protocol reported previously^27^. Briefly, images were acquired in the short and horizontal long-axis planes using a Siemens MAGNETOM Prisma 3.0T CMR with five-element phased array coil. Ungated, real-time steady-state free-precession cine imaging was performed at rest and during exercise using a supine cycle ergometer (MR Ergometer Pedal, Lode, Groningen, the Netherlands) at 60% of the power output achieved during the upright CPET as this was previously determined to approximate maximal supine exercise capacity^27,28^.

Images were analysed offline in RightVol (KUL, Leuven, Belgium) as previously described^27^. Stroke volume index (SVI) was calculated as end-diastolic volume minus end-systolic volume, indexed to body surface area, while cardiac index (CI) was calculated as the average of right- and left-ventricular stroke volume (RVSV + LVSV/2) multiplied by HR, indexed to body surface area. Left- and right-ventricular ejection fractions (LVEF, RVEF) were calculated as (SV/end-diastolic volume) multiplied by 100. Cardiac reserve was defined as the ability to augment CI from rest to peak exercise (peak CI minus resting CI). Arteriovenous oxygen difference (a-vO_2_ difference) was estimated via the Fick equation using CPET-derived ${{\dot{\mathrm V}}}$O_2peak_ (L min^−1^) and estimated upright CO_peak_ (ExCMR-derived supine SV_peak_ multiplied by  CPET-derived upright HR_peak_). Left and right end-diastolic and systolic volume reserve indexes (LVEDVI, RVEDVI, LVESVI, and RVESVI reserve, respectively) were calculated by subtracting resting EDV or ESV values from peak values, indexed to body surface area.

*Biochemistry.* Resting morning fasting blood samples were collected to measure hemoglobin, glucose and lipid profile, as well as the cardiac biomarkers, Troponin-I (cTn-I) and B-natriuretic peptide (BNP). All samples were analysed at the Alfred Health NATA-accredited pathology laboratory.

*Anthropometry and Body Composition.* Height (m) and body mass (kg) were measured via a fixed stadiometer (SECA, Hamburg, Germany) and portable scales (Coverall Medical Technologies, Victoria, Australia), respectively, and used to calculate body mass index and body surface area (DuBois Method). Total body lean mass (LM, kg), fat mass (FM, kg) and percent body fat (%BF) were quantified via dual-energy X-ray absorptiometry (GE Lunar iDXA, GE Healthcare, Little Chalfont, UK), and analysed using enCore software (version 14.10.022).

*Blood Pressure.* After 10-min of supine rest in a quiet room, brachial blood pressure and HR were measured using an automated oscillometric blood pressure monitor (OMRON HEM-907, OMRON Corporation, Tokyo, Japan). Blood pressure was defined as the average of three measurements made ≥ 3-min apart.

*Definitions.* ‘Cardiotoxicity’ was assessed using four different criteria. Echocardiographic criteria included LVEF < 50% and GLS >< −16% as per the European Society of Cardiology Position Statement of cancer treatment and cardiovascular toxicity^29^, and biochemical cut-points, specifically, BNP > 100 ng L^−1^ and cTn-I > 15 ng L^−1^, which represented the upper limit for our centre. Achievement of ≥ 1 of the above criteria signified cardiotoxicity. ‘Cardiac determinants’ of ${{\dot{\mathrm V}}}$O_2peak_ comprise cardiac biomarkers (cTn-I, BNP); echocardiography-derived indices (resting LVEF, GLS, HR); and CMR-derived indices (rest and exercise CI, SVI, HR, LVEF, RVEF). ‘Non-cardiac’ determinants of ${{\dot{\mathrm V}}}$O_2peak_ comprise hemoglobin concentration (hematological); resting blood pressure (vascular); CPET- and CMR-derived a-vO_2_ difference and DXA-derived indices (LM, FM, %BF) (skeletal muscle/body composition).

### Statistical analysis

Data were analysed using SPSS software (version 24.0, Statistical Package for the Social Sciences, IBM, Chicago, USA). The distribution of continuous variables was assessed using the Kolmogorov–Smirnov test. Continuous data are presented as mean ± standard deviation (SD), if normally distributed, or as median (interquartile range) for non-normally distributed variables. Categorical variables are presented as number (%). Differences in cardiovascular outcome measures were compared using an unpaired t-test or Mann–Whitney *U* test for continuous variables, and Fisher’s exact tests for dichotomous variables. Differences in the ExCMR cardiac response to exercise was assessed using repeated measures ANOVA, with Bonferroni correction for post-hoc analysis. Correlation analysis (Pearson’s for normally distributed variables or Spearman’s for non-normally distributed variables) were used to test for associations between organ-specific indices and ${{\dot{\mathrm V}}}$O_2peak_. A two-tailed *P* < 0.05 was considered statistically significant.

### Ethics approval

The study was approved by the Alfred Hospital Ethics Committee and conformed to the ethical standards set by the Helsinki Declaration.

### Consent to participate

Informed consent was obtained from all participants included in the study.

## Results

### Participant demographic, anthropometric and traditional cardiovascular risk characteristics

As shown in Table 1, the 14 long-term allo-SCT survivors and 14 non-cancer controls recruited to participate were matched for age and sex, and there were no significant differences in anthropometric variables. Seventy-nine percent of survivors had ≥ 1 traditional cardiovascular risk factor, compared to 43% of controls (*p* = 0.12) and a higher proportion of survivors tended to be treated for hypertension (29% vs 0%, *p* = 0.097). Two survivors had developed overt CVD and/or suffered a serious cardiovascular event after allo-SCT (heart failure, n = 1; symptomatic coronary artery disease and myocardial infarction, n = 1) (*p* = 0.48 compared to control).

**Table 1:** Demographic, anthropometric, and traditional cardiovascular risk characteristics of Allo-SCT survivors and Controls.

	Allo-SCT survivors	Controls	*P*
Age, years	44 ± 15	46 ± 13	0.75
Male sex, n (%)	7 (50)	7 (50)	> 0.99
Height, m	1.72 ± 0.95	1.73 ± 0.82	0.65
Weight, kg	72.3 ± 14.8	72.2 ± 15.4	0.99
Body mass index, kg.m^-2^	24.4 ± 3.9	23.9 ± 3.6	0.71
Body surface area, m^-2^	1.85 ± 0.23	1.85 ± 0.24	0.97
**Cardiovascular risk profile, n (%)**			
Overweight	8 (57)	6 (43)	0.71
Hypertension	4 (29)	0 (0)	0.097
Hyperlipidaemia	1 (7)	0 (0)	> 0.99
Diabetic	1 (7)	0 (0)	> 0.99
History of CVD Event	2 (14)	0 (0)	0.48
≥ 1 risk factor	11 (79)	6 (43)	0.12
**Cardiovascular medication, n (%)**			
Statin	1 (7)	0 (0)	> 0.99
ACE-I/ARB	3 (21)	0 (0)	0.22
Beta-blocker	1 (7)	0 (0)	> 0.99
Vasodilator	1 (7)	0 (0)	> 0.99

Data are mean ± SD or n (%); Abbreviations: *ACE-I*, angiotensin-converting-enzyme inhibitors; *ARB* angiotensin II receptor blockers; *CVD* cardiovascular disease.

### Prior cancer treatment and transplant related information for Allo-SCT survivors

Acute lymphoblastic leukaemia was the most common transplant indication (43%) and 93% of survivors had received anti-cancer therapy prior to receiving their allo-SCT (Table 2). Median time since transplantation was 6.5 years (range 2–20 years) and the most common donor type, graft source, conditioning intensity, conditioning regimen and GvHD prophylaxis were unrelated to donor, peripheral blood stem cells, myeloablative, cyclophosphamide/TBI (total body irradiation) and methotrexate/ciclosporin, respectively. Overall, 57% of survivors had a history of GvHD, affecting the gastrointestinal tract (n = 7, 50%), skin (n = 5, 36%), liver (n = 4, 29%), and/or lungs (n = 1, 7%).

**Table 2:** Prior cancer treatment and transplant related information for Allo-SCT survivors.

	N = 14
**Time since transplantation, years**	6.5 (2—20)
**Hematological malignancy, n (%)**	
Acute lymphoblastic leukaemia	6 (43)
Acute myeloid leukaemia	3 (21)
Non-Hodgkin lymphoma	2 (14)
Chronic lymphocytic leukaemia	1 (7)
Chronic myeloid leukaemia	1 (7)
Multiple myeloma	1 (7)
**Pre-transplant anti-cancer treatment, n (%)**	
No prior treatment	1 (7)
Chemotherapy	12 (86)
Immunotherapy	6 (43)
Targeted therapy	3 (21)
Autologous SCT	1 (7)
**Transplant donor, n (%)**	
Unrelated	8 (57)
Related	6 (43)
**Transplant graft source, n (%)**	
Peripheral blood	9 (64)
Bone marrow	5 (36)
**Transplant conditioning intensity, n (%)**	
Myeloablative	11 (79)
Reduced intensity	3 (21)
**Transplant conditioning regimen, n (%)**	
Cyclophosphamide/TBI	10 (71)
Fludarabine/Melphalan	1 (7)
Fludarabine/Melphalan/Campath	1 (7)
Fludarabine	1 (7)
Busulfan/Cyclophosphamide	1 (7)
**GvHD prophylaxis, n (%)**	
Methotrexate/ciclosporin	11 (79)
Post-transplant Cyclophosphamide/ciclosporin	1 (7)
Ciclosporin/mycophenolate	1 (7)
Ciclosporin	1 (7)
**History of GvHD, n (%)**	
No	6 (43)
Yes	8 (57)
**Length of hospital stay, days**	25 ± 4

Data are n (%), mean ± SD, or median (range). Abbreviations: *GvHD* graft-vs-host disease; *TBI* total body irradiation

### Integrative cardiovascular reserve capacity: cardiopulmonary exercise testing parameters

**Figure 1:**
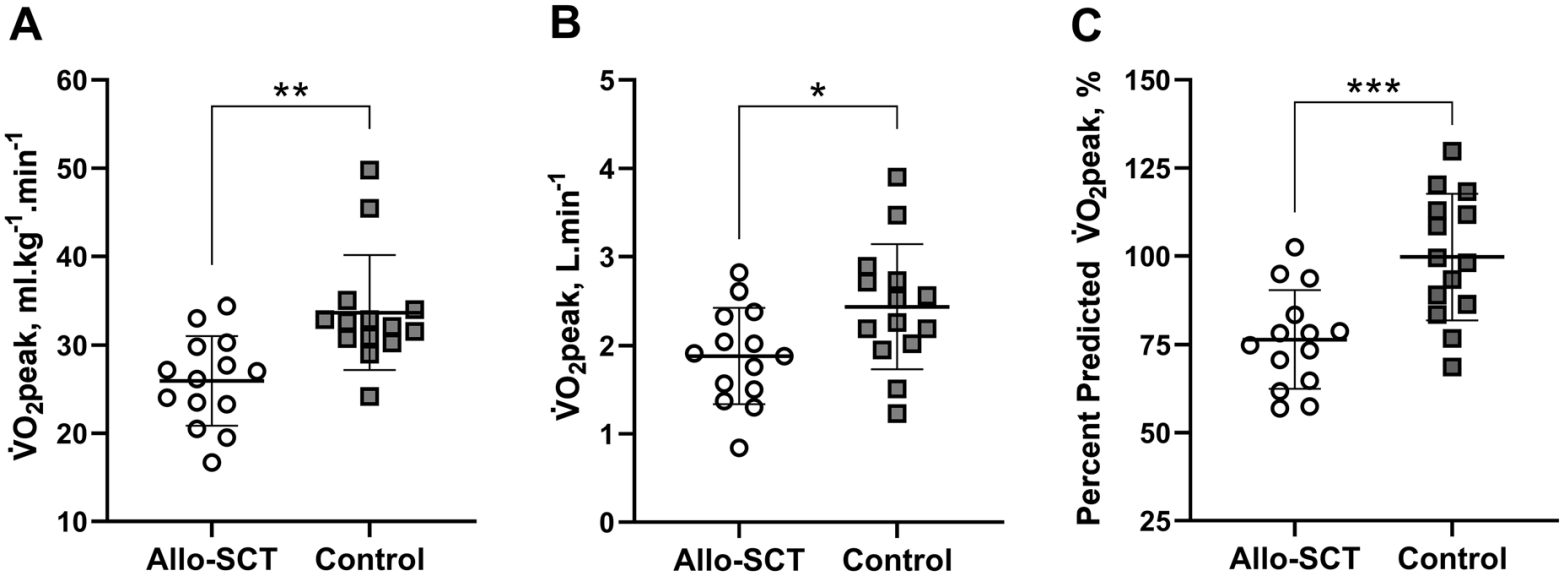
(**A**)–(**C**) Bodyweight-indexed (**A**), absolute (**B**) and percent-predicted ${{\dot{\mathrm V}}}$O_2peak_ (**C**) values for long-term Allo-SCT survivors and age- and sex-matched controls. Bodyweight-indexed, absolute and percent predicted ${{\dot{\mathrm V}}}$O_2peak_ were significantly lower in long-term allo-SCT survivors as compared with controls (*p* = 0.002, *p* = 0.027 and *p* < 0.001). Values are presented as mean ± SD and individual data points.

All participants reached criterion to indicate a peak exercise response to CPET. Differences in cardiopulmonary fitness parameters between survivors and matched non-cancer controls are summarised in Fig. 1 and Table 3. Survivors achieved a 23% lower absolute (*p* = 0.027) and bodyweight-indexed ${{\dot{\mathrm V}}}$O_2peak_ (*p* = 0.002) than controls. In the context of predicted ${{\dot{\mathrm V}}}$O_2peak_, this equated to 76% of predicted among survivors and 100% of predicted for the control group (*p* < 0.001). Survivors also achieved a lower peak power output (*p* = 0.016) and oxygen pulse (*p* = 0.019), and a trend toward lower SBP_peak_ (*p* = 0.069) compared with controls, but no group differences were observed for DBP_peak_ (Table 3). Arterial blood oxygen saturation was preserved in all participants during CPET.

**Table 3:** Comparison of peak cardiopulmonary exercise testing parameters between Allo-SCT survivors and Controls.

	Allo-SCT Survivors	Controls	*P*
${{\dot{\mathrm V}}}$˙O_2peak_, ml kg^-1 ^min^−1^	25.9 ± 5.1	33.7 ± 6.5	0.002
${{\dot{\mathrm V}}}$˙O_2peak_, L min^-1^	1.88 ± 0.54	2.44 ± 0.71	0.027
${{\dot{\mathrm V}}}$˙O_2peak_, % predicted	76 ± 14	100 ± 18	< 0.001
Peak power output, watts	176 ± 64	239 ± 66	0.016
HR_peak_, beats min^−1^	178 ± 15	176 ± 14	0.74
HR_peak_, % predicted	100 ± 6	100 ± 4	0.85
RER_peak_	1.34 ± 0.11	1.35 ± 0.14	0.82
Peak oxygen pulse, mL beat^−1^	10.9 ± 2.8	14.1 ± 3.8	0.019
SBP_peak_, mmHg	170 ± 30	190 ± 25	0.069
DBP_peak_, mmHg	88 ± 13	84 ± 10	0.39

Data are mean ± SD; Abbreviations: *DBP*_*peak*_ diastolic blood pressure, *HR*_*peak*_ peak heart rate, *RER*_*peak*_ peak respiratory exchange ratio, *SBP*_*peak*_ systolic blood pressure, ${{\dot{\mathrm V}}}$*O*_*2peak*_ peak oxygen uptake.

### Cardiac determinants of VO_2peak_

#### Resting cardiac function and biomarker profile

**Figure 2:**
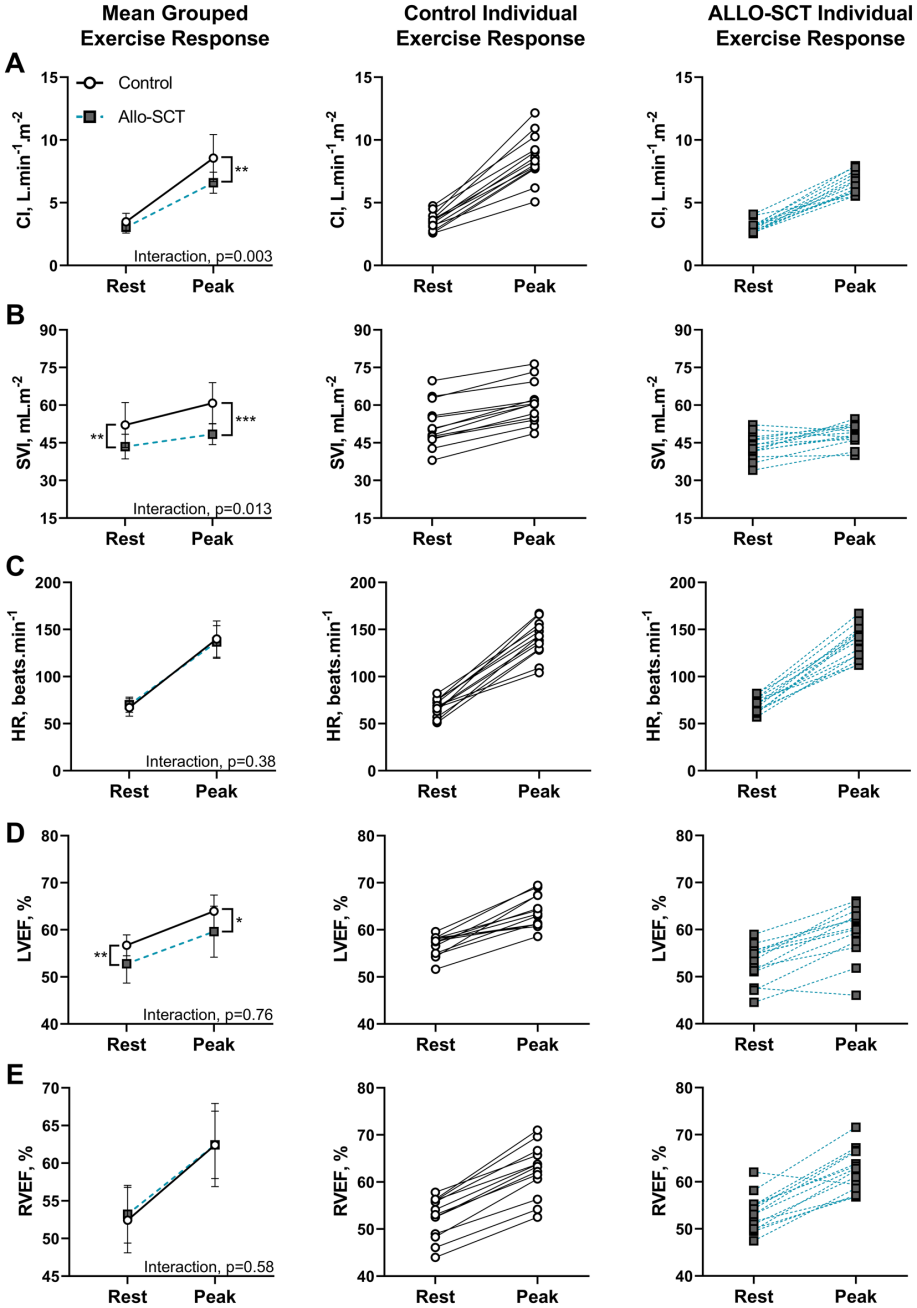
(**A**)–(**E**) Biventricular hemodynamic and ejection fraction response to supine cycling exercise in Allo-SCT survivors and Controls. Long-term survivors of Allo-SCT had reduced resting SVI, and a blunted augmentation in SVI and CI during exercise compared to controls (Panel A and B, respectively), whilst the HR response was similar (Panel C). LVEF and RVEF augmented similarly between groups (Panel D and E, respectively), although LVEF was lower at rest and peak exercise in Allo-SCT. Group data (left panels) is mean ± SD.

Results relating to resting cardiac function and biomarker profile are summarised in Table 4. There were no significant differences in echocardiography-derived LVEF or GLS nor blood-based markers of cardiac stress or injury between survivors and controls. The proportion of controls versus survivors with reduced LVEF (0% vs. 14%) or GLS (0% vs. 7%), or elevated cTn-I (13% vs. 7%) or BNP (0% vs. 7%) was similar (all *P* > 0.05; n = 2 allo-SCT met cardiotoxicity criteria and n = 2 control participants displayed similar characteristics). Regarding resting CMR-derived measures (Fig. 2A–E), resting RVEF and HR were similar between groups (*p* = 0.62 and *p* = 0.39, respectively), but resting LVEF and SVI were significantly lower in survivors (53 ± 4 vs 57 ± 2%, *p* = 0.005; 44 ± 5 vs 52 ± 9 ml m^−2^, *p* = 0.004) and there was a trend toward lower resting CI in survivors (3.1 ± 0.5 vs 3.5 ± 0.7 L min^−1^ m^−2^, *p* = 0.06).

**Table 4:** Comparison of resting cardiac function, biomarker profile, and biventricular diastolic and systolic reserve between Allo-SCT survivors and Controls.

	Allo-SCT survivors	Controls	*P*
**Cardiac biomarkers**			
cTn-I, ng L^−1^	2 (1 − 4)	2 (2 − 4)	0.52^a^
BNP, ng L^−1^	22 (15 − 37)	19 (14 − 36)	0.80^a^
**Resting echocardiography**			
LVEF, %	56 ± 6	59 ± 5	0.16
GLS, %	− 19 (− 18 − − 20)	− 20 (− 18 − − 21)	0.13^a^
HR, beats min^−1^	68 ± 8	63 ± 10	0.16
**ExCMR**			
LVEDVI reserve, mL m^−2^	− 1 ± 4	3 ± 3	0.006
RVEDVI reserve, mL m^−2^	− 4 ± 8	− 2 ± 7	0.46
LVESVI reserve, mL m^−2^	− 6 ± 3	− 5 ± 3	0.47
RVESVI reserve, mL m^−2^	− 8 ± 5	− 10 ± 5	0.35

Data are mean ± SD or median (IQR); Abbreviations: *BNP* B-natriuretic peptide, *cTn-I* cardiac troponin-I, *GLS* global longitudinal strain, *ExCMR* exercise cardiac magnetic resonance imaging, *HR* heart rate, *LVEDVI* left-ventricular end-diastolic volume index, *LVEF* left-ventricular ejection fraction, *LVESVI* left-ventricular end-systolic volume index, *RVEDVI* right-ventricular end-diastolic volume index, *RVESVI* right-ventricular end-systolic volume index.

^a^analysed using Mann–Whitney *U* test.

#### Cardiac reserve

Results relating to the biventricular hemodynamic and ejection fraction response to supine cycling exercise are shown in Table 4 and Fig. 2A–E. Survivors had a blunted increase in CI from rest to peak exercise (interaction, *p* = 0.003), such that CI_peak_ was 23% lower than controls (6.6 ± 0.8 vs 8.6 ± 1.9 L min^−1^ m^−2^; *p* = 0.001). This difference was due to between group differences in SVI augmentation (interaction, *p* = 0.013) given that the HR response was similar (*p* = 0.38). SVI_peak_ was 21% lower in survivors than controls (48 ± 4 vs 61 ± 8 ml m^−2^; *p* < 0.001), which was predominantly due to a blunted LVEDVI reserve (*p* = 0.006), as RVEDVI and biventricular ESVI reserve were not different between groups. LVEF and RVEF augmented similarly in response to exercise in survivors and controls (interactions, *p* = 0.76 and *p* = 0.58, respectively), but LVEF was lower in survivors at rest (53 ± 4 vs 57 ± 2%, *p* = 0.005) and peak exercise (60 ± 5 vs 64 ± 3%, *p* = 0.021).

### Non-cardiac determinants of ${{\dot{\mathrm V}}}$O_2peak_ and blood-based metabolic risk profile

Differences in non-cardiac contributors to ${{\dot{\mathrm V}}}$O_2peak_ and blood-based metabolic risk profile between survivors and controls are summarised in Table 5. Despite similar bodyweight and body mass index, FM was on average 5.8 kg higher (*p* = 0.069) and LM 4.3 kg lower (*p* = 0.25) in survivors compared to controls, resulting in a significantly greater total %BF in allo-SCT (mean difference, 7.9%, *p* = 0.007). No group differences were observed for hemoglobin, blood pressure, peak a-vO_2_ difference or blood-based metabolic risk profile.

**Table 5:** Comparison of non-cardiac determinants of ${{\dot{\mathrm V}}}$O_2peak,_ and blood-based metabolic risk profile between Allo-SCT survivors and Controls.

	Allo-SCT survivors	Controls	*P*
**Non-cardiac determinants**			
Hemoglobin, g L^−1^	140.2 ± 12.5	141.8 ± 9.6	0.71
Total LM, kg	44.7 ± 7.9	49.0 ± 10.8	0.25
Total FM, kg	26.1 ± 8.9	20.3 ± 7.3	0.069
Body fat percentage, %	35.7 ± 7.1	27.8 ± 7.1	0.007
SBP, mmHg	127 ± 15	117 ± 15	0.11
DBP, mmHg	76 ± 10	70 ± 7	0.10
a-vO_2_ difference, mL dL^−1^	11.7 ± 2.1	12.3 ± 1.5	0.34
**Blood-based metabolic risk profile**			
Total cholesterol, mmol L^−1^	5.1 ± 0.9	5.1 ± 0.9	0.95
Triglycerides, mmol L^−1^	1.6 ± 0.7	1.2 ± 0.7	0.12
HbA1c, %	5.4 ± 0.2	5.3 ± 0.4	0.28

Data are mean ± SD; Abbreviations: *a-vO2 difference* arteriovenous oxygen difference, *DBP* diastolic blood pressure, *FM* fat mass, *HbA1c* glycated haemoglobin, *LM* lean mass, *SBP* systolic blood pressure

### Associations between ${{\dot{\mathrm V}}}$O_2peak_ and central and peripheral organ function in long-term allo-SCT survivors

Among long-term allo-SCT survivors, ${{\dot{\mathrm V}}}$O_2peak_ (L min^−1^) was strongly (positively) associated with measures of exercise cardiac function (CO_peak_: r = 0.91, R^2^ = 0.83, *p* < 0.001; SV_peak_: r = 0.77, R^2^ = 0.60, *p* = 0.001), LM (r = 0.65, R^2^ = 0.42, *p* = 0.012), hemoglobin (r = 0.61, R^2^ = 0.38, *p* = 0.020) and peak a-vO_2_ difference (r = 0.78, R^2^ = 0.61, *p* = 0.001) (see supplementary Fig. 1 for correlation plots). There were no significant associations between ${{\dot{\mathrm V}}}$O_2peak_ and FM (r = 0.22, R^2^ = 0.05, *p* = 0.45), %BF (r =  − 0.29, R^2^ = 0.09, *p* = 0.31), resting SBP (r =  − 0.10, R^2^ = 0.01, *p* = 0.74), resting DBP (r = 0.10, R^2^ = 0.01, *p* = 0.72), LVEF (r = 0.16, R^2^ = 0.03, *p* = 0.58), GLS (Spearman r =  − 0.04, *p* = 0.89), resting HR (r =  − 0.2, R^2^ = 0.04, *p* = 0.48), HR_peak_ (r = 0.26, R^2^ = 0.07, *p* = 0.36), cTn-I (Spearman r = 0.19, *p* = 0.54) or BNP (Spearman r = 0.20, *p* = 0.49).

## Discussion

The main finding from this matched case–control cross-sectional study, which is the first study to combine integrated measures of functional capacity with measures of cardiac function during exercise in survivors of allo-SCT, was that cardiovascular reserve was markedly impaired in survivors as measured by ${{\dot{\mathrm V}}}$O_2peak_, an integrated measure of exercise capacity, or measured specifically as cardiac reserve by ExCMR. On the other hand, despite a less favourable body composition and higher prevalence of hypertension among survivors, peripheral muscle oxygen extraction at peak exercise (peak a-vO_2_ difference) was similar between survivors and non-cancer age- and sex-matched controls suggesting that reductions in central cardiac delivery of oxygen may be the primary explanation for functional impairment in allo-SCT survivors. However, our results do lend support to the possibility of impairment in diffusive oxygen conductance (peripheral limitation) among survivors given the similar peak a-vO_2_ difference despite lower CI_peak_.

In the present study, long-term survivors of allo-SCT achieved a ${{\dot{\mathrm V}}}$O_2peak_ that was, on average, 23% lower than age- and sex-matched controls and 24% below predicted values. This deficit is of clinical significance as cardiovascular reserve capacity is finite in nature, and continued pathological perturbations seen with age, inactivity or additional cancer therapy culminate in overt cardiovascular dysfunction^24^. Specifically, data from the general population and patients with cancer indicates that with every 3.5 ml kg^−1^ min^−1^ decrement in ${{\dot{\mathrm V}}}$O_2peak_ there is a corresponding increased risk of incident heart failure (16–21%), and all-cause (25–26%), cardiovascular- (14%) and cancer-related mortality (25%)^18,30,31^. Therefore, with the 7.8 ml kg^−1^ min^−1^ deficit in ${{\dot{\mathrm V}}}$O_2peak_ observed among the survivors in our study it is not unexpected that this population are burdened by markedly elevated rates of overt CVD, and premature cardiovascular and all-cause mortality. Our results are consistent with Armenian et al.^21^ who found ${{\dot{\mathrm V}}}$O_2peak_ to be 22% below predicted values at a median of 9.8 years post-SCT, but intriguingly, are in contrast to the degree of ${{\dot{\mathrm V}}}$O_2__peak_ impairment observed among short, and *very* long-term survivors^13,22^. Indeed, ${{\dot{\mathrm V}}}$O_2peak_ has been shown to be 49% below predicted among early survivors (~ 3 months post-SCT)^13^ and 11% below predicted among *very* long-term survivors of allo-SCT (median 17 years [range 6–26] post-SCT)^22^. It may therefore be reasonable to speculate that allo-SCT-induced impairments in ${{\dot{\mathrm V}}}$O_2peak_ ‘recover’ to some degree over time. However, this optimistic interpretation may rather be explained by the ‘healthy survivor’ bias wherein allo-SCT recipients with particularly poor cardiovascular health (and thus, low ${{\dot{\mathrm V}}}$O_2peak_) may have died prematurely. Prospective, longitudinal studies with periodic assessment are needed to elucidate this further.

Impairments in ${{\dot{\mathrm V}}}$O_2peak_ can result from cardiac, pulmonary, vascular, hematologic, or skeletal muscle limitations to oxygen delivery transit and metabolism. Thus, delineating the origin(s) of dysfunction in this cohort is important to help inform therapeutic approaches to ameliorate these effects. To date, there has been limited study of cardiac function and cardiac reserve in the context of allo-SCT. To address this knowledge gap, we employed a combination of conventional and contemporary tools to characterise cardiac function. A key finding of our study was that despite marked differences in ${{\dot{\mathrm V}}}$O_2peak_, there were no differences in standard-of-care resting echocardiographic measures of cardiac function nor sensitive blood-based biomarkers of cardiac stress or injury between survivors and controls, which remained, on average, within normal ranges. Furthermore, we found no associations between measures of resting cardiac function and ${{\dot{\mathrm V}}}$O_2peak_. In contrast, utilising novel, state-of-the-art ExCMR, we were able to unmask impairments in cardiac function among survivors, evidenced by a blunted CI reserve which was secondary to a blunted SVI reserve and not chronotropic impairment. Further, several survivors showed minimal augmentation of SVI, or even a decline in SVI during exercise (Fig. 2B, Allo-SCT individual exercise responses). These maladaptive myocardial responses to exercise are likely a consequence of direct (exposure to cardiotoxic anti-cancer treatments, steroid use) and indirect (physical inactivity, prolonged sedentary time) treatment-related insults which can detrimentally alter ventricular systolic and/or diastolic function^29,32–35^. Interestingly, while some survivors exhibited myocardial exercise responses consistent with systolic dysfunction (impaired ventricular contractility), it appears diastolic limitations (impaired ventricular filling) may be the predominant mediator of the blunted SVI reserve among this cohort of long-term survivors—evidenced by a significantly blunted EDVI reserve (driven by LVEDVI) and similar biventricular ESVI reserve. Whether this is reflective of impaired ventricular relaxation and/or an increase in ventricular stiffness is unclear and warrants further investigation. Moreover, ExCMR measures of exercise cardiac function were strongly, and positively correlated with ${{\dot{\mathrm V}}}$O_2peak_, offering a likely explanation for the impairments in ${{\dot{\mathrm V}}}$O_2peak_ seen in allo-SCT survivors. These results, indicating impaired cardiac reserve, are consistent with our observations in early survivors^13^ and support the increasingly accepted notion that assessing cardiac function under the hemodynamic and metabolic stress of exercise may be more sensitive to ascertaining heart failure risk than conventional resting assessment when the metabolic demands are low^24^. Taken together, we demonstrate that survivors of allo-SCT exhibit persistent subclinical myocardial dysfunction that partly explains their ${{\dot{\mathrm V}}}$O_2peak_ impairment, which we speculate explains their higher rates of CVD, increased morbidity, and premature mortality.

Given survivors of allo-SCT are subjected to multiple cardiovascular insults (chemotherapy, radiotherapy, targeted therapy, immunotherapy, GvHD) and lifestyle perturbations (deconditioning, malnutrition) throughout the course of their treatment journey^36^, we anticipated a multifactorial contribution to ${{\dot{\mathrm V}}}$O_2peak_ impairment. This notion is supported in part by our recent work^13^ and others^37–39^ in early allo-SCT survivors demonstrating impaired skeletal muscle oxygenation and/or pulmonary function and their positive association with low exercise capacity. Metrics of skeletal muscle oxygenation have not been previously characterised in the longer-term survivor, but Myrdal et al.^22^ similarly reported a 44% prevalence of impaired pulmonary diffusion capacity among *very* long-term survivors which was significantly associated with low ${{\dot{\mathrm V}}}$O_2peak_. However, conflicting results from Armenian et al.^21^ who reported no association between pulmonary indices and VO_2peak_ among long-term survivors, and the present study wherein arterial blood oxygen saturation was preserved and peak a-vO_2_ difference was similar between our control and long-term allo-SCT survivor cohort suggests a multifactorial contribution to ${{\dot{\mathrm V}}}$O_2peak_ impairment may not always be the case. Indeed, limitations of pulmonary origin may be directly dependent on the development of pulmonary complications such as lung GvHD (prevalence 40%^22^ vs. 0%^21^ vs. 7% in our study). Regarding peak a-vO_2_ difference, it is worth noting, however, that in the context of reduced blood flow to skeletal muscle (e.g., lower CI_peak_) as seen in our survivor cohort, there is effectively more time for oxygen diffusion, and thus the similar ratio of oxygen extraction (peak a-vO_2_ difference) infers an impairment in diffusive oxygen conductance which could suggest reduced capillary to fiber ratio, altered capillary dynamics or impairments in oxygen utilisation in muscle reducing the oxygen diffusion gradient. This observation warrants further investigation and validation. Moreover, long-term allo-SCT survivors also displayed a less than favourable body composition, evidence by a significantly higher %BF related to lower total body LM (− 4.3 kg) and higher FM (+ 5.8 kg) and were also more likely to be treated for hypertension (29% of survivors vs. 0% controls). These are common features of aging which may predispose allo-SCT survivors to serious cardiovascular sequelae and ultimately, premature CVD. Indeed, the risk of serious post-SCT cardiovascular complications is reported to increase incrementally with increasing cardiovascular risk factors^6,10^, and a suboptimal LM to FM ratio can potentiate CVD via common pathogenic pathways including insulin resistance, dyslipidaemia, and inflammation^40^. Overall, while the marked impairment in ${{\dot{\mathrm V}}}$O_2peak_ may not be explained by impairments in skeletal muscle oxygen extraction capabilities per se, the long-term allo-SCT survivors in this study showed signs of accelerated cardiovascular and skeletal muscle aging which warrant surveillance.

The results of the present study are hypothesis-generating, ultimately shedding light on potential opportunities for therapeutic avenues to ameliorate the deleterious effects of allo-SCT (and prior treatment) on cardiac reserve, body composition profile, and subsequently ${{\dot{\mathrm V}}}$O_2peak_ and cardiovascular risk. From a pharmacological standpoint, cardiovascular medications such as beta-adrenergic receptor agonists, angiotensin-converting enzyme inhibitors, angiotensin receptor antagonists and calcium channel blockers have the theoretical potential to improve/recover SVI reserve (via enhancing myocardial relaxation and/or ventricular compliance) and therefore ${{\dot{\mathrm V}}}$O_2peak_ in survivors^41^. Though, initiation of cardioprotective pharmacotherapy for subclinical cancer-therapy related cardiac dysfunction is not yet routinely indicated due to ongoing debates over risk–benefit ratio (especially in those with LVEF ≥ 50%)^42–45^. Moreover, non-pharmacological approaches such as exercise training—which is safe, has low barriers for initiation (i.e., low cost), targets the entire oxygen transport utilisation pathway (and thus ${{\dot{\mathrm V}}}$O_2peak_)^46–49^, and can favourably modulate body composition profile^29,50^—may represent an effective therapeutic strategy in this population wherein polypharmacy and financial burden is high^51,52^. Further, dietary modification (in accordance with established cardiovascular disease prevention nutrition guidelines^53–55^) is an effective strategy for improving traditional cardiovascular risk and body composition profile, and when combined with exercise, may enhance its beneficial effects on cardiac function and VO_2peak_. Notably, exercise training and nutritional counselling constitute core components of cardiovascular rehabilitation for individuals with established CVD^46,47,55–59^, and our group recently demonstrated the utility of combined aerobic and resistance exercise in improving ${{\dot{\mathrm V}}}$O_2peak_ and cardiac reserve (via increased SV reserve) among anthracycline-treated breast cancer patients^60^. Taken together, there is strong theoretical rationale for further investigation into the therapeutic utility of exercise training, nutritional counselling and/or cardioprotective pharmacotherapy to improve ${{\dot{\mathrm V}}}$O_2peak_, cardiac reserve and body composition profile among allo-SCT survivors.

There are a few limitations that require consideration when interpreting the findings of this study. The small sample size precluded sub-analyses of potential treatment-related modifiers of ${{\dot{\mathrm V}}}$O_2peak_ (e.g., conditioning intensity; history of GvHD; anthracycline, cyclophosphamide, and radiation dose; steroid use) and increases the likelihood of a type II error. The cross-sectional design introduces potential survivor bias wherein our survivor cohort may be ‘healthier’ than the typical allo-SCT recipient, and this would serve to underestimate the functional impairment in the typical allo-SCT survivor. While the impaired ${{\dot{\mathrm V}}}$O_2peak_ among survivors does not appear to be mediated by the pulmonary system (reflected by preserved arterial blood oxygen saturation during CPET), the lack of direct pulmonary evaluation in this study (via spirometry, pulmonary CT) limits our ability to definitively rule out the presence of subtle functional and/or structural pulmonary maladaptation that could progress to compromise arterial blood oxygen saturation, and therefore ${{\dot{\mathrm V}}}$O_2peak_, over subsequent years. In addition, due to the innate ability of the peripheral muscle to compensate for upstream impairment (i.e., vascular), future studies should extend our work to better describe vascular health and its relationship with ${{\dot{\mathrm V}}}$O_2peak_ in this population using more sensitive measures of vascular aging (e.g., arterial compliance, arterial elastance, and endothelial function). Finally, indirect determination of a-vO_2_ difference has its limitations due to the assumptions required for its calculation (utilisation of supine SV to estimate upright CO). Further, the Fick equation itself may be a simplification when discerning peripheral defects as it does not capture oxygen diffusion limitations. This simplification is because a-vO_2_ difference (as determined by the Fick Equation) reflects a ratio but does not indicate the absolute oxygen diffusion into muscle which interacts with blood flow/oxygen delivery as outlined above. It is therefore important to acknowledge that peripheral defects as defined by the Fick principle (i.e., a-vO_2_ difference) are not interchangeable with peripheral defects defined by the Fick law of diffusion—as illustrated by Howden et al.^61^. Consequently, the possibility of impairment in peripheral diffusive conductance (i.e., diffusion capacity) should not be discounted and warrants further investigation^62,63^.

In conclusion, long-term survivors of allo-SCT exhibit a marked impairment in cardiovascular reserve capacity (${{\dot{\mathrm V}}}$O_2peak_) compared to age- and sex-matched counterparts, and this deficit appears to be largely attributed to subclinical myocardial dysfunction. In addition, long-term survivors exhibit a poorer body composition profile as reflected by the higher %BF which was driven by lower LM and higher FM. Taken together, our results (i) highlight the utility of subclinical CVD assessment (specifically exercise cardiovascular assessment) in unmasking cardiovascular dysfunction, (ii) draw attention to the persistent nature of cardiovascular dysfunction in this population, and (iii) provide valuable insight into the pathophysiologic mechanisms driving premature CVD. Each of the identified deficits can be responsive to therapy, especially exercise training. Thus, our results identify pathology that can be targeted to mitigate cardiovascular burden in allo-SCT survivors.

## Supplementary Information

Supplementary Information 1.

## Data Availability

The datasets generated during and/or analysed during the current study are available from the corresponding author on reasonable request.
